# Biological function and molecular mechanism of piRNA in cancer

**DOI:** 10.1016/j.plabm.2018.e00113

**Published:** 2018-12-07

**Authors:** Ghanbar Mahmoodi Chalbatani, Hassan Dana, Feridon Memari, Elahe Gharagozlou, Shirin Ashjaei, Peyman Kheirandish, Vahid Marmari, Habibollah Mahmoudzadeh, Farnaz Mozayani, Ali Reza Maleki, Ehsan Sadeghian, Elham Zainali Nia, Seyed Rohollah Miri, Neda zainali Nia, Omid Rezaeian, Anahita Eskandary, Narges Razavi, Mohammad Shirkhoda, Fatemeh Nouri Rouzbahani

**Affiliations:** a Cancer Research Center, Cancer Institute of Iran, Tehran University of Medical Science, Tehran, Iran; b Department of Paramedical Sciences, Islamic Azad University, Tehran Branch, Tehran, Iran; c Department of Biology, Damghan Branch, Islamic Azad University, Damghan, Iran; d Department of Biology, Tonekabon Branch, Islamic Azad University, Tonekabon, Iran; e Department of Biology, NourDanesh Institute of Higher Education, Isfahan, Iran; f Shahid Beheshti University of Medical Sciences, Tehran, Iran

**Keywords:** Cancer, PiRNA, Noncoding RNA, Gene silencing, RNAi

## Abstract

Cancer is the second leading cause of death globally. piRNAs, which are a novel type of identified small noncoding RNA (ncRNA), play a crucial role in cancer genomics. In recent years, a relatively large number of studies have demonstrated that several piRNA are aberrantly expressed in various kinds of cancers including gastric cancer, bladder cancer, breast cancer, colorectal cancer and Lung cancer and may probably serve as a novel therapeutic target and biomarker for cancer treatment. The present review summarized current advances in our knowledge of the roles of piRNAs in cancer.

## 1 Introduction

Irrespective of socio-economic context, Cancer is the second leading cause of death globally, since it is the third leading cause of death in low- and middle-income countries and the second leading cause of death in high-income countries [1], [2], [3], [4].

SncRNAs (Small non-coding RNAs) are part of non-coding oligonucleotide regulators with wide morphologic and physiologic functions. At the transcriptional and post-transcriptional levels, these molecules are primary mediators of the gene regulation [5]. sncRNAs have a variety of family members, among which the most investigated are small nucleolar RNAs, small nuclear RNAs, siRNA (small interfering RNA) [6], miRNAs (micro-RNAs) [7], and piRNAs (PIWI-interacting RNAs) [8].

**Fig. 1:**
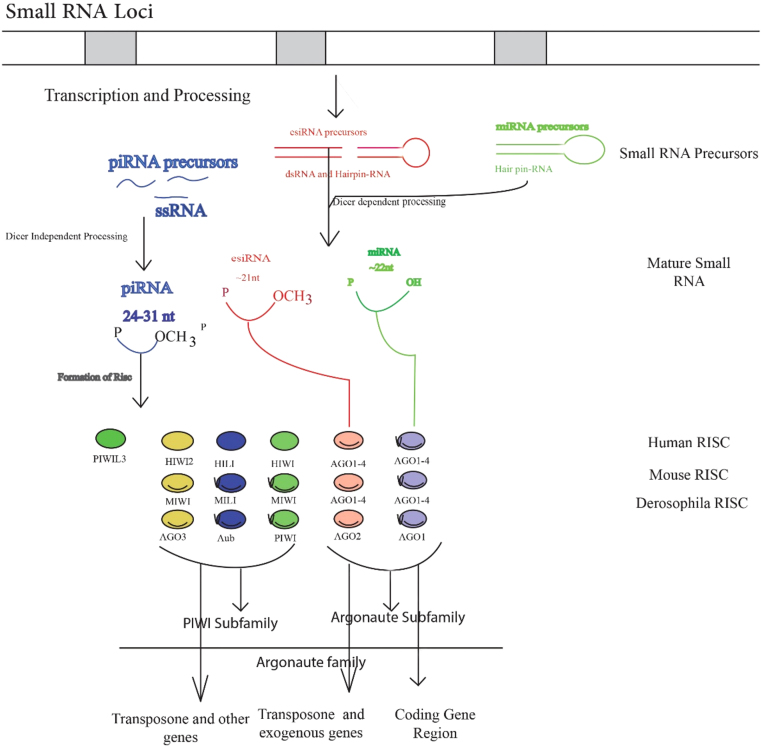
RNA silencing by small RNAs and their partner Argonaute family proteins in Drosophila, human, and mouse RNA. The expression level of 4 PIWI protein (PIWIL3) has been a discovery in humans. The mains roles of the mature sequences, RISC formation, piRNA precursors and target genes are summarized for miRNAs, and piRNAs. The correlation between piRNA and human PIWI protein has not yet been detect. Abbreviations: dsRNA, double-stranded RNA; esiRNA, endogenous small interfering RNA(siRNA); miRNA, microRNA; nt, nucleotide; piRNA, PIWI-interacting RNA; RISC, RNA-induced silencing complex; ssRNA, single-stranded RNA.

RNAi (RNA interference), also denoted as RNA silencing, in most eukaryotes has emerged as one of the key gene regulatory pathways [9], [10]. Central to RNAi pathways is the generation of small RNAs of 20–31 nt (nucleotides). These small RNA, produced by a processing protein known as Dicer or by Dicer-independent processes, form complexes with RISC (RNA-induced silencing complex) carrying Argonaute proteins [11], [12]. RISCs are directed to the target genes based on the complementarities between target gene transcripts and small RNAs and inhibit their expression by RNA instability, by inducing translational inhibition or by cleaving the transcripts [13], and/or heterochromatinization [14], [15]. miRNAs and endogenous short interfering RNAs (endo-siRNAs) associate with the Argonaute (AGO) subfamily members, while piRNAs produced by a Dicer-independent process associate with PIWI subfamily members of proteins [16] (Fig. 1).

piRNAs are a class of sncRNA molecules that have been recently recognized to be relevant to cancer biology. More than 30,000 piRNAs have been found in humans [17], [18]. They are best characterized by their role in guiding associated chromatin-silencing machinery to transposon-encoding DNA sequences in the genome [18], [19]. Researchers have offered that a subset of piRNAs may also be capable of regulating protein-coding genes via DNA methylation, which, if the regulatory targets are cancer-relevant, may bear on cancer development [20], [21], [22].

piRNAs independent of RNase III enzymes are generated from single-stranded precursors in a manner [23], [24], [25], [26] and they are as usual 26–31 nt (nucleotides) long. The mechanisms underlying piRNA functions and biogenesis mainly remain unknown, mostly because the piRNA pathway has little in common with the miRNA and endo-siRNA pathways as well as the restriction of piRNA territories to the reproductive tissues [23].

The piRNAs are generally processed from their longer precursors that are transcribed from introns, 3′-UTR regions and repetitive elements [27], [28]. They are a distinct class of small RNAs that function in transposon silencing, epigenetic regulation, and germline development [29], [30], [31]. These molecules have remarkable diversity, with tens of thousands of unique sequences in mammals and over 1.5 million unique sequences in Drosophila [32], [33].

## 2 Methodology

In this review, we analyze the recent research on the piRNA in cancer biology also, we summarized some of the sever cancer, which happens in the human body.

## 3 The role of piRNAs in cancer

The functions of PIWI proteins and piRNAs have started to emerge in human cancers [34]. A growing number of evidences have found that PIWI proteins in mice and humans such as PIWIL2-like proteins, HIWI and PIWIL2 are expressed in various types of tumor cells [35], [36]. Furthermore, piRNAs were also detected in these cells [36]. The deregulated expression of piRNAs has been reported in human cancers, including gastric cancer, bladder cancer, breast cancer, colorectal cancer and Lung cancer. These findings indicate that the piRNA pathway may be linked to cancer development. Though the potential role of piRNAs in cancer has just emerged and remains to be investigated, but, functional role of specific piRNAs is poorly understood in human cancer. These results highlight the importance of understanding the exact role of the piRNA pathway during tumorigenesis and offered new possibilities for tumor therapy.

**Table:** 

piRNA	Expression		Cancer	Function	Reference
	UP	Down			
piR−34736		*	Breast	Induced by cell cycle progression	[37]
piR−36249		*	Breast	Induced by cell cycle progression	[37]
piR−35407		*	Breast	Induced by cell cycle progression	[37]
piR−36318		*	Breast	Induced by cell cycle progression	[37]
piR−34377		*	Breast	Induced by cell cycle progression	[37]
piR−36743	*		Breast	Induced by cell cycle progression	[37]
piR−36026	*		Breast	Induced by cell cycle progression	[37]
piR−31106	*		Breast	Induced by cell cycle progression	[37]
piRABC		*	Bladder	Increase the expression of TNFSF4 protein	[38]
PiR−823		*	Gastric	Inhibit cancer cell growth	[39]
piR−55490		*	Lung	Suppress the activation of Akt/mTOR pathway by binding 3^/^UTR of mTOR messenger RNA and induce its degradation	[40]
piR-L−163		*	Lung	Bind directly to phosphorylated ERM protein ) p-ERM)	[41]
piR-Hep1	*		Liver	deep sequencing of cell lines, validated in matched tumor-normal tissues via PCR	[42]
piR−015551		*	Colorectal	Associated with recurrence-free survival	[43]

### 3.1 Gastric cancer

Gastric cancer is the second most significant reason for global cancer-related mortalities [44], [45] and the fifth most prevalent cancer in the world [46], [47]. In the United States, there are about 7.4 new cases of gastric cancer per 100,000 women and men per year [48]. Also in 2016, with 26,420 deaths and 42,280 new cases, esophageal and gastric cancers rank among the deathliest malignant diseases in the U.S.A [49]. Based on a research in Europe, 4 of 5 patients with gastric cancer die within the first 5 years after diagnosis [50].

piR-823 was downregulated in gastric cancer tissue with no association between its clinicopathological features and expression levels [51]. Most importantly, the growth of gastric cancer cells was inhibited by piR-823 mimics in vitro and tumor growth in vivo (in a xenograft model) was significantly suppressed, both in a dose-dependent manner. These results, suggesting that piR-823 is a possible therapeutic target and tumor suppressor for Gastric cancer. piR-651 as an oncogene was overexpressed in gastric cancer, resulting in a positive correlation with TNM (tumor node metastasis) stage. This observation was consistent with results in other cancer tissues and cell lines such as breast, lung, colon and liver cancers. Of course, the research has not shown that the expression of piR-651 was associated with other clinicopathological findings such as sex, age, invasion and tumor size. In addition, in the G2/M phase, a piR-651 inhibitor could inhibit cell growth, which was an important indication that piRNAs play a crucial role in tumorigenesis [52]. In addition, piR-651 and piR-823 were both reported to be at lower levels in CTCs (circulating tumor cells), compared with normal controls, in peripheral blood of gastric cancer patients [53]. However, both piR-823 and piR-651 than the commonly used biomarkers such as CA19-9 (carbohydrate antigen 19–9) and CEA (serum carcinoembryonic antigen) for gastric cancer were more sensitive because as a short fragment, piRNAs are not so easily degraded, and levels of piR-651 as well as piR-823 in blood samples are relatively stable and these piRNAs can pass through the cell membrane, and can be detected and isolated easily from body fluids. It has been also suggested that gastric cancer patients from healthy controls can be detected, by way of measuring the levels of piR-823 and piR-651 in peripheral blood, making possible an early diagnosis of gastric cancer. These findings suggest that piRNAs could be novel therapeutic targets in gastric cancer [54].

### 3.2 Breast cancer

In the United States of America, there are around 3.1 million breast cancer survivors. The chance of any woman dying from breast cancer is around 2.7%, or 1 in 37. 710 new diagnoses of breast cancer are expected in women and 610 women are likely to die from the disease in 2017. Deep sequencing was carried out in four matched non-tumor tissues and four breast cancer tissues, to screen out differentially expressed piRNAs. Afterwards, by RT-PCR in 50 breast cancer, 4 piRNAs (piR-20365, piR-20582, piR-20485 and piR-4987) were confirmed to be up-regulated. The clinical pathology feature of patients, such as: estrogen receptor (ER) status, tumor size Her2 status and lymphnode status were recorded. Also the up-regulation of piR-4987 was positively associated with lymph node metastasis [55]. In a study it was showed that piR-932 /PIWIL2 complex through promoting the methylation of Latex may positively regulate the process of breast cancer stem cells, which in turn promotes EMT (epithelial-mesenchymal transition). It has been suggested that both PIWIL2 and piR-932 could be potential targets for blocking the metastasis of breast cancer [56]. Similarly, another study found that piR-021285 is involved in methylation at a number of known breast cancer-related genes, in particular, attenuated 5′ untranslated region (UTR)/first exon methylation at the proinvasive ARHGAP11A gene and invasiveness in an in vitro cell line model [57], [58].

### 3.3 Bladder cancer

Bladder cancer in the world is the 9th most common malignancy and the most common malignancy of the urinary tract [59], [60]. In the United States of America, from bladder cancer are expected an estimated 74,690 new cases and 15,580 deaths [61]. Using the ArrayAtarHG19 piRNA array, for 23,677 human piRNAs, the researchers profiled three pairs of bladder cancer tissues and their adjacent normal tissues. They identified piRABC (also called DQ594040) as a relevant piRNA being down-regulated in bladder cancer [62]. piRABC showed very high differential expression levels between normal tissues and bladder cancer. Studies in vitro on human bladder cancer cell lines suggested that the overexpression of piRABC may inhibit the promotion of cell apoptosis, cell proliferation and colony formation. Researchers showed a possible interaction with Tumor Necrosis Factor Superfamily Member 4 (TNFSF4) hypothesizing that piRABC may promote Bladder Cancer cell apoptosis by up regulation of TNFSF4 [63].

### 3.4 Lung cancer

Lung cancer is broadly divided into non-small cell lung cancer (approximately 85% cases) and small cell lung cancer (approximately 15% cases) as well as it’s the leading cause of cancer-related death in the world [64]. Researcher demonstrated that piRNAs are expressed in somatic HBE (human bronchial epithelial) cells, and the expression patterns are distinctive between lung cancer cells and normal bronchial epithelial cells [64]. Furthermore, they have shown that piR-L-163 could directly bind and regulate phosphorylated ERM and play a critical role in protein activation [65], [66]. The researchers identified that piR-L-138 was upregulated upon cisplatin (CDDP)-based chemotherapy both in vivo and in vitro, and that targeting it could be a potential strategy to overcome chemoresistance in patients with lung squamous cell carcinoma (LSCC) [67].

### 3.5 Liver cancer

Liver cancer is the second most common cause of cancer death for men and women combined worldwide [68]. Liver cancer is the 5th most usual cancer among men and the 9th most common cancer among women and although it occurs more frequently in less developed regions of the world but it is still a significant health outcome in the United States of America [69]. The of using combined biological and bioinformatics analyses, a number of studies have identified and characterized less well-explored non-coding RNAs in human hepatocellular carcinoma (HCC) [70], [71]. Law et al. identified piR-Hep1, be upregulated in nearly half of the HCC tumors (46.6%) compared to their corresponding adjacent non-tumoral liver. Silencing of this piRNA inhibited invasiveness, cell viability and motility with a concomitant reduction in the level of active AKT phosphorylation [72], [73], [74].

### 3.6 Colorectal cancer

Colorectal cancer is the third most common cancer in men and the second in women worldwide [75], [76]. Some researchers have observed that PIWI contributes to the development of colorectal cancer [77], [78]. Chu et al proposed that piRNAs through binding to PIWI may play an important role in the risk of colorectal cancer. So, they proposed that piRNAs through binding to PIWI may play an important role in the risk of colorectal cancer and also hypothesized that genetic variants in piRNAs could modulate colorectal cancer susceptibility [79]. Yin et al. proposed that piRNA-823 was one of the piRNAs which contributed to colorectal carcinogenesis. They observed that knocking down this piRNA could suppress induce G1 phase arrest and cellular apoptosis, the viability of colorectal cancer cells. They suggested that piRNA-823 could be a potential therapeutic target for colorectal cancer.

## 4 Conclusion

There are many studies reporting the involvement of non-coding RNAs in cancer progression and development such as siRNAs, and miRNAs, but piRNAs have only recently been identified as new prognostic and diagnostic tools.

According to the functions of piRNAs such as transcriptional and post-transcriptional regulatory, which are certainly not restricted to silencing transposable elements, they seem to present great potential for future interventions in the course of diseases, including cancer and also provide new insights into cancer epigenetics. Investigation into the role of piRNAs in the establishment of transcriptional patterns —and their involvement in key processes in germline and epigenetic, genetic and recently, somatic cells—is a rapidly growing field of research.

## 5 Recommendations

Understanding of piRNAs complex interactions will provide many novel interventions either medical discovery and in clinical practice or in biological, improving the understanding and management of cancer. The abovementioned studies indicate that investigations focus on exploration of the importance of piRNAs in cancer. Therefore, this research in the future is likely to result in better treatment strategies based on these piRNAs interactions.
